# The impact of threat of shock-induced anxiety on memory encoding and retrieval

**DOI:** 10.1101/lm.045187.117

**Published:** 2017-10

**Authors:** Sorcha Bolton, Oliver J. Robinson

**Affiliations:** Institute of Cognitive Neuroscience, University College London, London WC1N 3AR, United Kingdom

## Abstract

Anxiety disorders are the most common mental health disorders, and daily transient feelings of anxiety (or “stress”) are ubiquitous. However, the precise impact of both transient and pathological anxiety on higher-order cognitive functions, including short- and long-term memory, is poorly understood. A clearer understanding of the anxiety–memory relationship is important as one of the core symptoms of anxiety, most prominently in post-traumatic stress disorder (PTSD), is intrusive reexperiencing of traumatic events in the form of vivid memories. This study therefore aimed to examine the impact of induced anxiety (threat of shock) on memory encoding and retrieval. Eighty-six healthy participants completed tasks assessing: visuospatial working memory, verbal recognition, face recognition, and associative memory. Critically, anxiety was manipulated within-subjects: information was both encoded and retrieved under threat of shock and safe (no shock) conditions. Results revealed that visuospatial working memory was enhanced when information was encoded and subsequently retrieved under threat, and that threat impaired the encoding of faces regardless of the condition in which it was retrieved. Episodic memory and verbal short-term recognition were, however, unimpaired. These findings indicate that transient anxiety in healthy individuals has domain-specific, rather than domain-general, impacts on memory. Future studies would benefit from expanding these findings into anxiety disorder patients to delineate the differences between adaptive and maladaptive responding.

Anxiety disorders are debilitating mental health conditions that constitute an emotional, social, and economic burden (Collins et al. 2011). The impact that anxiety has on cognition is a principal contributing factor to this, particularly within the domain of memory. One of the core symptoms of post-traumatic stress disorder (PTSD), for instance, is intrusive reexperiencing of a traumatic event in the form of vivid memories (National Collaborating Centre for Mental Health 2005). Furthermore, it has been suggested that anxiety disorder patients may selectively retrieve past information, which perpetuates their negative beliefs about a current or imagined situation (Zlomuzica et al. 2014), and it has been demonstrated that individuals with high levels of trait anxiety have facilitated memory for self-threatening information (Saunders 2013).

However, clinical understanding of memory alterations in anxiety disorders is largely derived from subjective self-report measures. This is problematic because an individual's pattern of behavioral responses on cognitive tasks is a far more reliable proxy of memory performance than self-report (Shanks and John 1994; Vadillo et al. 2016). However, studies directly exploring the impact of anxiety disorders on experimental tasks have yielded mixed results. Some studies have found anxiety disorder patients exhibit impairments in short-term verbal and visual memory (Jelinek et al. 2006; O'Toole et al. 2015), including facial recognition (Jarros et al. 2012), and long-term memory (Airaksinen et al. 2005; Butters et al. 2011). Other studies show no anxiety disorder-linked impairment in short-term (Günther et al. 2004; Castaneda et al. 2011) or recognition memory (Yoon et al. 2016).

One reason for these discrepancies might be that anxiety in patient populations is often concomitant with other psychiatric or physical illnesses, and there are often wide variations both in terms of disorder onset and medication history. Changes to cognitive functioning may therefore be unrelated to anxiety (Guez et al. 2016). To gain a better experimental insight into how cognitive processes are disrupted by certain aspects of anxiety we can explore the effects of “inducing” anxiety in healthy individuals.

Anxiety has been operationalized as a response to prolonged, unpredictable threat (Robinson et al. 2013b). Threat of unpredictable electric shock therefore provides a robust, translational method of inducing anxiety within-subjects: individuals are either at risk of receiving a mild electric shock, or safe from shock. This measure can be thought of as inducing a normal, “adaptive” anxiety response. This type of anxiety is adaptive because it primes defensive responses to promote harm avoidance (Robinson et al. 2013b). Nevertheless this manipulation has reliable psychological, psychophysiological, and neural effects that mimic symptoms seen in anxiety disorders (Robinson et al. 2012, 2013b, 2014; Aylward and Robinson 2016). Moreover, this method presents a further methodological advantage with regard to the study of memory, as it allows anxiety to be independently manipulated at both memory encoding and retrieval. This is important as it has been suggested that differences in the time at which anxiety is induced (i.e., during memory encoding versus retrieval) may contribute to the equivocal findings regarding the relationship between anxiety and memory (Het et al. 2005; Robinson et al. 2013b). Additionally, using an experimental design can help shed light on whether memory impairments may be a cause or a consequence of anxiety.

In this study, we therefore explore the impact of induced anxiety on the encoding and retrieval stages of (1) visuospatial working memory (spatial span task), (2) verbal short-term recognition memory, (3) face recognition memory, and (4) episodic/associative memory. With the aim of assessing the impact of anxiety on a broad range of memory processes, tasks that are commonly used in the literature, and are well validated, were chosen for the present study.

## Anxiety and short-term verbal and visuospatial memory

Short-term (working) memory (WM) can be thought of as a system that temporarily stores and manipulates a limited amount of information (Moran 2016). In general terms, it has been suggested that working memory (WM) is restricted by anxiety, as anxiety is thought to compete with task-relevant processes (Stefanopoulou et al. 2014). Broadly speaking, the model of WM that is most commonly encountered in anxiety research separates WM into verbal and visuospatial domains: we will use this distinction in the present study. Research has consistently demonstrated that threat of shock impairs spatial and verbal WM performance on the N-Back task (Lavric et al. 2003; Shackman et al. 2006; Vytal et al. 2012, 2013, 2016; Patel et al. 2016; Balderston et al. 2017b). However, generalization of N-Back findings onto other tasks assessing short-term memory is unclear. Moreover, findings suggest that both visuospatial and verbal N-back tasks have insufficient reliability, for example, compared with span tasks, making them insensitive to individual differences in working memory (Jaeggi et al. 2010). The current study addresses this limitation by investigating the impact of threat of shock on a short-term verbal recognition and spatial span task.

## Anxiety and face recognition

Facial recognition dysfunction may contribute to avoidance behaviors and atypical social interaction in anxiety disorders (particularly social anxiety disorders) (Gentili et al. 2016). Yet anxiety's relationship to facial recognition has not been comprehensively investigated (Yoon et al. 2016), and no known studies have investigated the impact of threat of shock on face recognition. Findings suggest that induced anxiety (CO_2_ inhalation) at the point of retrieval impairs facial recognition accuracy (Attwood et al. 2015), and Moon et al. (2016) demonstrated that threat (induced by affective pictures) immediately prior to retrieval, impaired facial recognition in anxious patients compared with controls. In both studies, this effect was only explored on retrieval. The current study aims to extend these findings, by examining the effect of threat of shock, at both encoding and retrieval, on facial recognition.

## Anxiety and episodic memory

Long-term memory refers to the storage of information over extended periods of time. It is commonly subdivided into; explicit memory, which includes semantic (general knowledge) and episodic (specific event) memory; and implicit memory, which is generally unconscious and involves memories about body movements (Tulving and Schacter 1990; Squire and Zola-Morgan 1991). Associative memory is a fundamental feature of episodic memory; it refers to the combining of different representations of an event, such as objects or the event's location, into a coherent whole (O'Keefe and Nadel 1979; Cohen and Eichenbaum 1993; Davachi 2006; Eichenbaum et al. 2007). Paradigms assessing associative memory are thought to be more ecologically valid than those assessing item memory (i.e., recall/recognition of an object), as accurate episodic memory involves retrieving information about the associations between the people and objects involved, not just the individual elements themselves. Indeed, evidence indicates that these processes are dissociable at both the behavioral (Jacoby 1991; Yonelinas 2002) and neural level (Aggleton and Brown 1999; Davachi 2006; Eichenbaum et al. 2007). The literature would therefore benefit from a more comprehensive investigation of the effects of anxiety on item and associative memory independently; the present studies aims to address this.

Meta-analytic results suggest that induced anxiety impairs explicit memory retrieval (Sauro et al. 2003). However, Het et al. (2005) argue that timing is important: anxiety induced prior to retrieval impairs performance, whereas anxiety before encoding seems to have little effect. Yet other studies using anxiety-inductions, such as the cold pressor test (submerging the participant's hand in ice water), have found impairments at both encoding and retrieval (Kuhajda et al. 1998; Savić et al. 2005; Ishizuka et al. 2007). These mixed findings may be due to the relative inefficacy of some anxiety-inductions, as anxiety is often induced pretask, with assessment timed to uncertain cortisol response peaks. This suggests that these inductions are not optimal for modelling anxiety-related impairments. To address this, the present study uses threat of shock (which induces a stress response on a much faster timescale) to assess the impact of stress on episodic (specifically, associative) memory, at both encoding and retrieval.

## The current study

In this study, participants will encode information under both threat and safe conditions, and then retrieve information under threat of shock versus safe conditions: enabling us to disentangle, within-subjects, the impact of anxiety on memory formation and retrieval.

This study was preregistered via the Open Science Framework (osf.io/zjpm2) and aims to test the following hypothesis: threat of shock impairs memory encoding and retrieval across all tasks, such that accuracy will be greatest in the safe-encoding/safe-retrieval condition, compared with the safe-encoding/threat-retrieval, threat-encoding/safe-retrieval, and the threat-encoding/threat-retrieval conditions. The mixed findings in the extant literature do not provide a clear rationale to predict domain-specific impairments, therefore it is posited that anxiety will impair all memory processes.

## Results

### Manipulation check

As shown in Table 1, participants reported feeling significantly more anxious when at threat compared with when safe: *P*-values were all <0.001 and logBF_10_ values were all >30 rejecting the null.

**Table 1.**
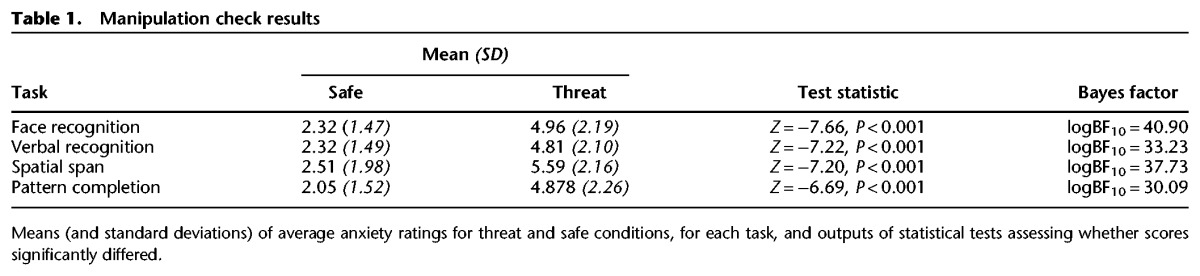
Manipulation check results

### Spatial span task

Two participants did not complete the spatial span task due to a technical fault, so *N* = 84.

#### Proportion correct

Scores across all conditions were non-normal and so were squared prior to analysis. Participants achieved a significantly higher proportion of correct responses when they encoded information during threat (*M* = 0.707, SD = 0.141) compared with safe (*M* = 0.682, SD = 0.128); significant main effect of encoding condition, *F*_(1,83)_ = 6.66, *P* = 0.012, η_*p*_^2^ = 0.074. There was also a significant main effect of retrieval condition, *F*_(1,83)_ = 5.82, *P* = 0.018, η_*p*_^2^ = 0.066, indicating that participants performed better when retrieving information under conditions of threat (*M* = 0.705, SD = 0.133), compared with under safe conditions (*M* = 0.684, SD = 0.131). These effects were qualified by a significant interaction between encoding and retrieval conditions, *F*_(1,83)_ = 12.41, *P* < 0.001, η_*p*_^2^ = 0.130.

**Figure 1.**
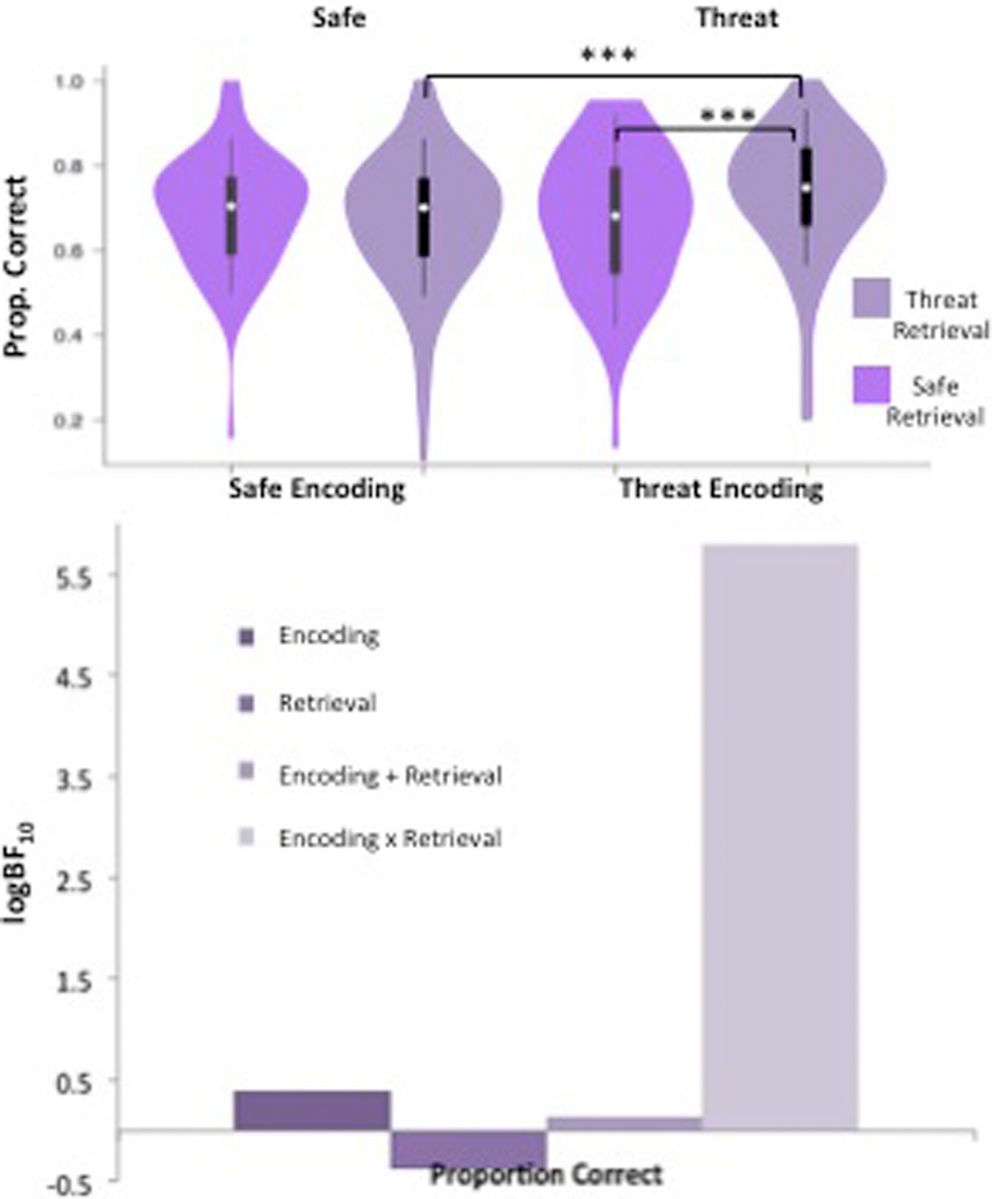
Spatial span task results. Violin plots displaying participants’ average anxiety ratings (for safe versus threat) and proportion correct for each condition, and bar charts representing the Bayesian model evidence for proportion correct for each condition. For the violin plots (and all those subsequent), the density estimates of data are shown; white circles represent the medians; boxes indicate the 25th/75th percentiles with whiskers extending ×1.5 the interquartile range.

Simple main effects analyses revealed that when information was retrieved under threat, proportion correct was significantly greater when information had been encoded during conditions of threat (i.e., in the threat/threat condition; *M* = 0.738, SD = 0.146) compared with during safe conditions (i.e., the safe/threat condition; *M* = 0.671, SD = 0.155), *F*_(1,83)_ = 18.20, *P* < 0.001, η_*p*_^2^ = 0.180. Furthermore, when information was encoded under threat, proportion correct was significantly greater at threat-retrieval (i.e., the threat/threat condition; *M* = 0.738, SD = 0.147) compared with safe-retrieval (i.e., the threat/safe condition; *M* = 0.675, SD = 0.163), *F*_(1,83)_ = 18.09, *P* < 0.001, η_*p*_^2^ = 0.179. These differences can be seen in Figure 1.

Bayesian analysis also confirmed a winning model comprising the encoding × retrieval interaction (BF_10_ = 266), which was decisively better than the null; a model including the main effects of encoding and retrieval without the interaction (BF_10_ = 2.83); an encoding alone model (BF_10_ = 2.88); and a retrieval alone model (BF_10_ = 0.90).

### Verbal recognition task

#### Proportion correct

**Figure 2.**
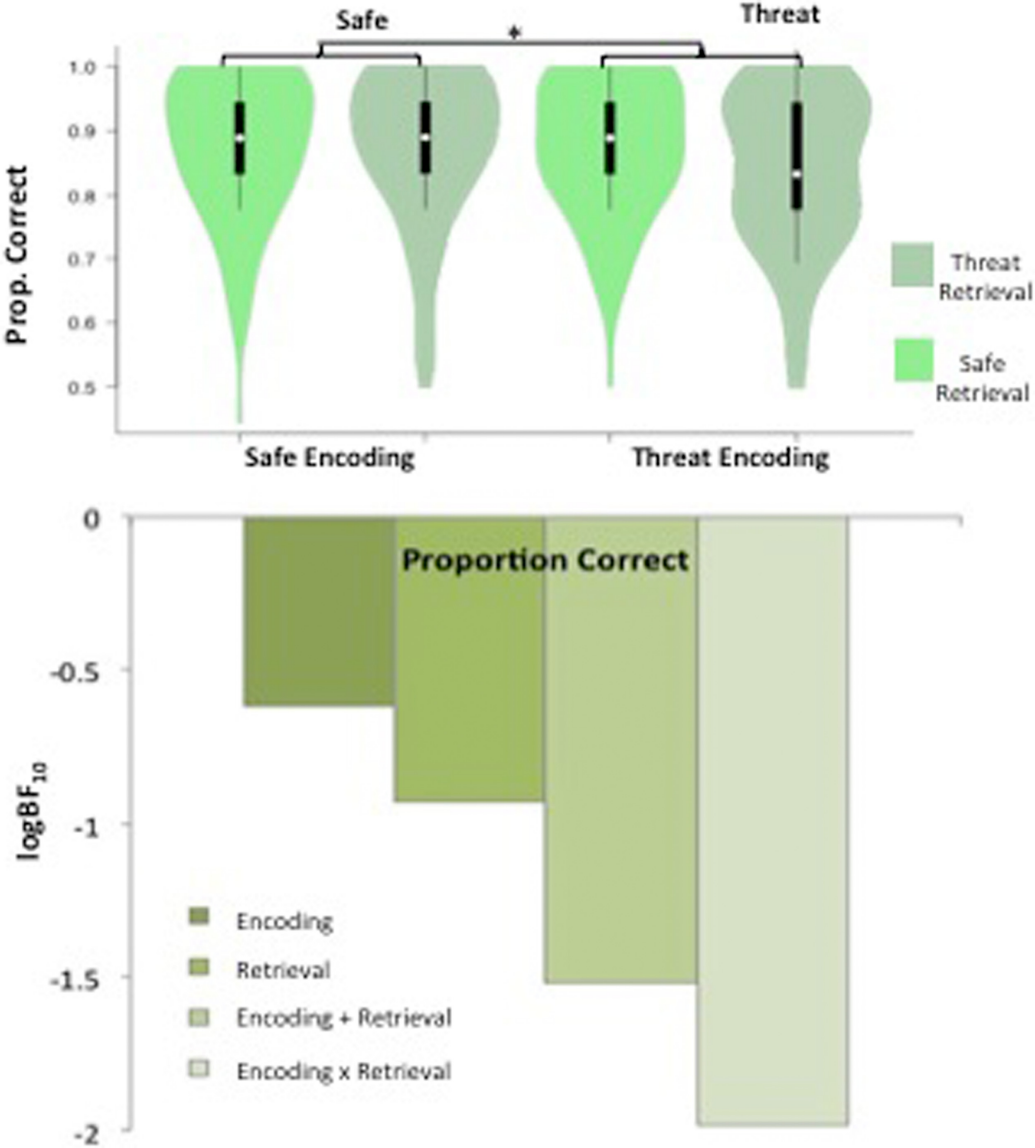
Verbal recognition task results. Violin plots displaying proportion correct for each condition, and bar charts representing the Bayesian model evidence for proportion correct for each condition.

Scores were non-normal and so were cubed prior to analysis to give a more normal distribution. There was a significant main effect of encoding condition, *F*_(1,85)_ = 4.02, *P* = 0.048, η_*p*_^2^ = 0.045; indicating that participants achieved a higher proportion of correct responses when they encoded information under conditions of safety (*M* = 0.872, SD = 0.099) compared with threat (*M* = 0.854, SD = 0.098) (Fig. 2). There was no significant main effect of retrieval condition, *F*_(1,85)_ = 2.19, *P* = 0.143, η_*p*_^2^ = 0.025, and no significant interaction between encoding and retrieval conditions, *F*_(1,85)_ = 2.64, *P* = 0.108, η_*p*_^2^ = 0.030.

However, Bayesian analysis did not provide support for the main effect of encoding condition; BF_10_ values were all <1, which provides evidence in favor of the null model. The null model was anecdotally (1.2 and 2.6 times, respectively) better than a model including the main effect of encoding only (BF_10_ = 0.80), and the main effect of retrieval only (BF_10_ = 0.33), and substantially (6.6 times) better than a model including the encoding × retrieval interaction (BF_10_ = 0.15).

#### Confidence

Scores were approximately normal and so analysis was run using the original data. Results revealed no significant main effect of encoding, *F*_(1,85)_ = 3.19, *P* = 0.078, η_*p*_^2^ = 0.036, or retrieval, *F*_(1,85)_ = 3.28, *P* = 0.074, η_*p*_^2^ = 0.037. There was no significant interaction between encoding and retrieval, *F*_(1,85)_ = 0.04, *P* = 0.847, η_*p*_^2^ < 0.001.

This pattern of results was confirmed by Bayesian analysis: the winning model was the null as all other BF_10_ values were <1. The null model was anecdotally better than models including the main effect of encoding only (BF_10_ = 0.50) and the main effect of retrieval only (BF_10_ = 0.69), and substantially better than a model including the encoding × retrieval interaction (BF_10_ = 0.155).

### Face recognition task

#### Proportion correct

**Figure 3.**
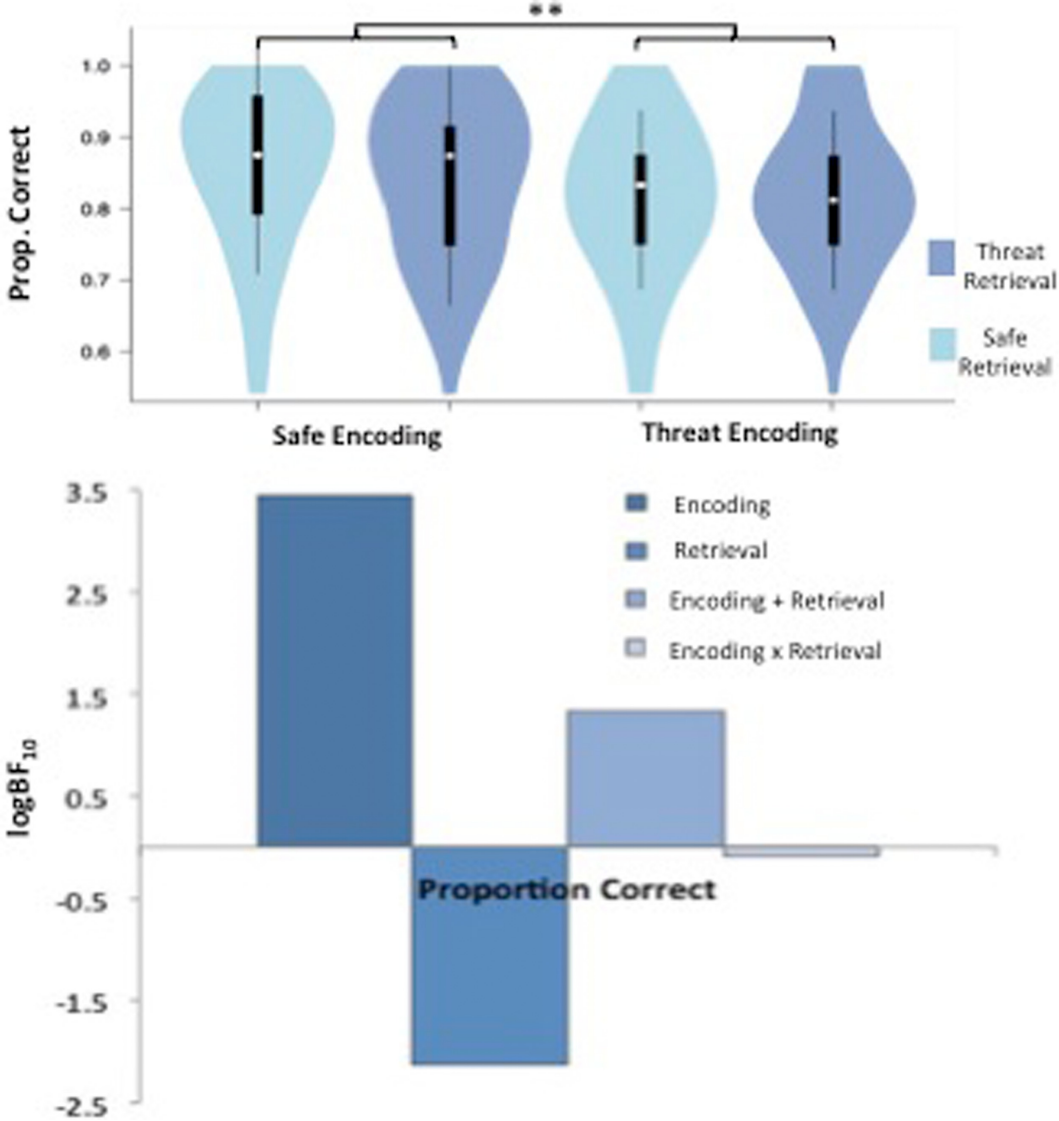
Facial recognition task results. Violin plots displaying proportion correct for each condition, and bar charts representing the Bayesian model evidence for proportion correct for each condition.

Scores were non-normal, and so were squared prior to analysis to give a more normal distribution. There was a significant main effect of encoding condition, *F*_(1,85)_ = 10.70, *P* = 0.002, η_*p*_^2^ = 0.112, because participants achieved a higher proportion of correct responses when they encoded information under conditions of safety (*M* = 0.846, SD = 0.097) compared with threat (*M* = 0.814, SD = 0.093) (Fig. 3). There was no significant main effect of retrieval condition, *F*_(1,85)_ = 0.19, *P* = 0.666, η_*p*_^2^ = 0.002, and no significant interaction between encoding and retrieval conditions, *F*_(1,85)_ = 0.82, *P* = 0.367, η_*p*_^2^ = 0.010.

Bayesian analysis confirmed a winning model comprising the main effect of encoding condition (BF_10_ = 52.68). This model was substantially better than a model additionally including the main effect of retrieval condition (BF_10_ = 6.99), and strongly better than a model additionally including the encoding × retrieval interaction (BF_10_ = 1.63).

#### Confidence

Scores were approximately normal and so analysis was run using the original data. Mirroring task performance, subjects were significantly more confident when information was encoded under safe (*M* = 5.994, SD = 1.40) compared with threat (*M* = 5.599, SD = 1.25), main effect of encoding condition: *F*_(1,85)_ = 9.03, *P* = 0.003, η_*p*_^2^ = 0.096. There was no significant main effect of retrieval condition, *F*_(1,85)_ = 0.46, *P* = 0.497, η_*p*_^2^ = 0.005, and no significant interaction between encoding and retrieval conditions, *F*_(1,85)_ = 0.01, *P* = 0.926, η_*p*_^2^ = 0.000.

Bayes factor analysis confirmed this finding, with the winning model comprising the main effect of encoding only (BF_10_ = 8.72). This model was substantially better than a model additionally including the main effect of retrieval (BF_10_ = 1.39), and strongly better than a model additionally including the encoding × retrieval interaction (BF_10_ = 0.22).

### Associative memory task

Eight participants did not complete the associative memory task due to a technical fault, so *N* = 78.

#### Proportion of correct associations

Proportion correct for each association was averaged across cue type. Therefore, the scores used in analysis represent the average proportion correct (per participant) when cued with a location, object, or person, for both safe and threat at encoding. Scores were approximately normal and so analysis was run using the original data.

**Figure 4.**
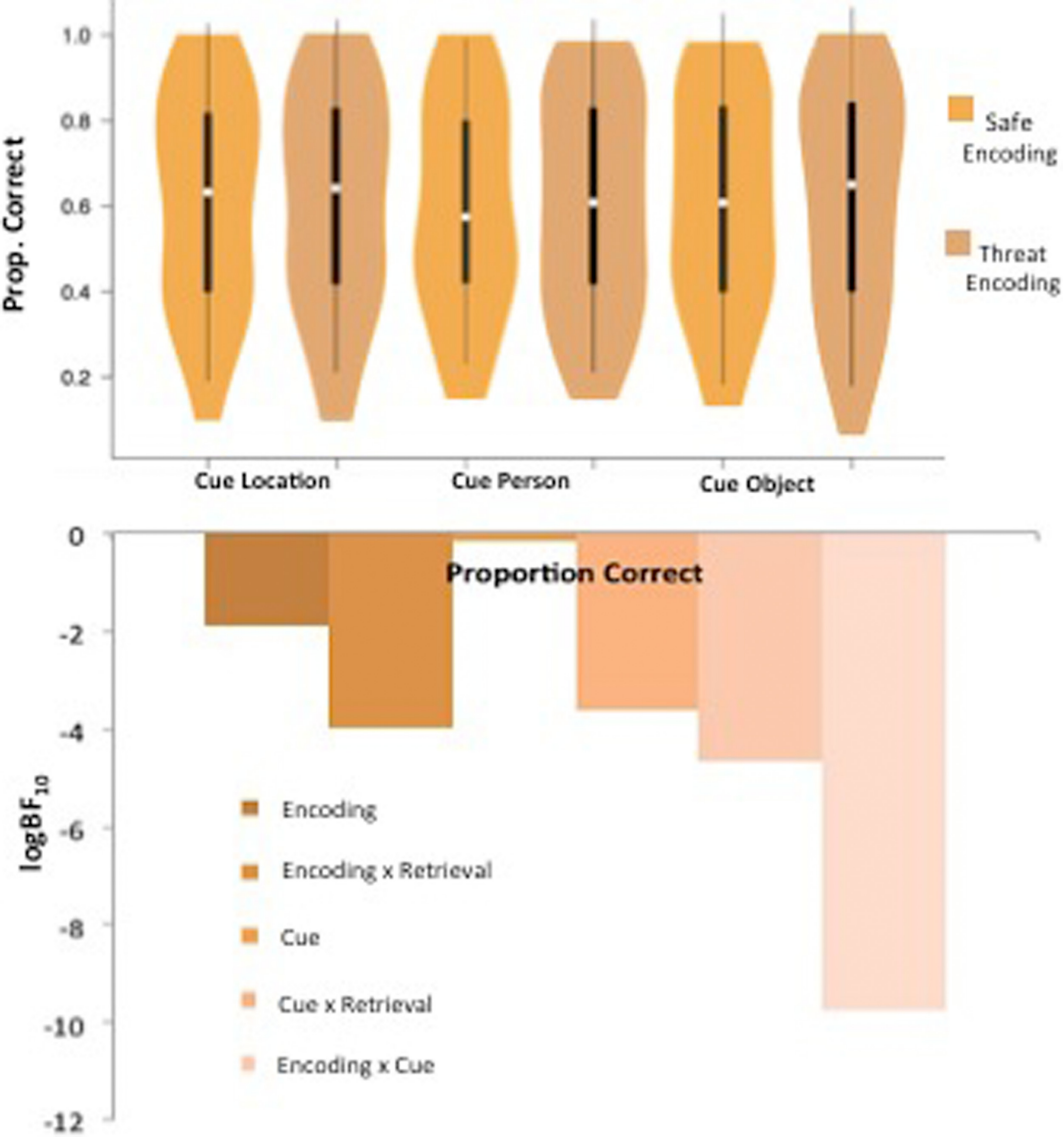
Associative memory task results. Violin plots displaying and proportion correct for each condition, and bar charts representing the Bayesian model evidence for proportion correct for each condition.

Results revealed no significant main effect of encoding condition, *F*_(1,76)_ = 0.624, *P* = 0.432, η_*p*_^2^ = 0.008, and no significant interaction between encoding and retrieval conditions, *F*_(1,76)_ = 0.436, *P* = 0.511, η_*p*_^2^ = 0.006; Figure 4. For cue type, Mauchly's test was significant and so the Huynh–Feldt correction was applied to correct for the violation of the sphericity assumption. There was a significant main effect of cue type, *F*_(1.83,138.68)_ = 3.61, *P* = 0.033, η_*p*_^2^ = 0.045; proportion correct was greater when participants were cued with a location (*M* = 0.612, SD = 0.250) or object (*M* = 0.611, SD = 0.254) compared with person (*M* = 0.596, SD = 0.245). There was no significant interaction between cue type and retrieval condition, *F*_(1.83,138.68)_ = 0.081, *P* = 0.908, η_*p*_^2^ = 0.001, and no significant interaction between cue type and encoding condition, *F*_(2,152)_ = 0.89, *P* = 0.413, η_*p*_^2^ = 0.012. There was a significant three-way interaction between encoding condition, cue type, and retrieval condition, *F*_(2,152)_ = 4.22, *P* = 0.016, η_*p*_^2^ = 0.053.

However, this significant three-way interaction was not followed up as Bayesian analysis found the winning model to be the null: all models had BF_10_ values <1. The null model was anecdotally (1.2 times) better than the main effect of cue type alone model (BF_10_ = 0.816), and decisively (>150 times) better than the encoding condition × retrieval condition × cue type model (BF_10_ = 5.826 × 10^−5^).

## Discussion

This study aimed to elucidate the effects of induced anxiety on memory encoding and retrieval. Partially consistent with predictions, threat was found to impair the encoding, but not retrieval, of faces. However, in the reverse of what was predicted, visuospatial WM was enhanced rather than impaired when information was encoded and subsequently retrieved under conditions of threat. Further inconsistent with hypotheses, threat had no significant effect on verbal short-term recognition memory or associative memory accuracy, at encoding or retrieval.

### Visuospatial working memory

Inconsistent with predictions, spatial span performance was enhanced when information was encoded and subsequently retrieved under conditions of threat. This contradicts previous research that demonstrated spatial WM was impaired by threat during the spatial N-back task (e.g., Vytal et al. 2013). Importantly however, a recent meta-analysis found that the N-Back task and measures of WM span are only modestly correlated: suggesting that these tasks measure different underlying WM processes (Redick and Lindsey 2013).

Pertinent to interpreting the current findings is the theory of context-dependent memory (Godden and Baddeley 1975) or mood-congruency; which posits that the recall of information is improved when the context present at retrieval (in this case, external conditions of threat versus safe; internal conditions of stress versus less stress) matches the encoding context (Murnane et al. 1999). However, if our pattern of results were solely a result of context-dependent memory then we would also expect to see improved performance when information was encoded and subsequently retrieved under conditions of safety. As performance was enhanced only in the threat-encoding/threat-retrieval condition, the findings must specifically be a result of anxiety rather than simply due to reencountering the encoding context. This idea of threat-specific context-dependent retrieval draws parallels with PTSD symptomatology: memories encoded in trauma situations (i.e., under threat) are vividly reexperienced when encountering environments that are reminiscent of the initial trauma (Wegerer et al. 2013). However, having said this, intrusive reexperiencing of memories in PTSD is not solely due to encountering environments reminiscent of the initial trauma; it is also thought to be a result of inappropriate memory cues. In other words, when information encoded as part of a traumatic event is reencountered (even under conditions of no anxiety/safety), this cues the reexperiencing of the initial trauma. Consequently, a parallel can be drawn between PTSD and the threat-encoding/safe-retrieval condition. As results revealed no significant differences in performance during this condition, to explore this relationship further, future work would benefit from more fully investigating visuospatial WM performance in populations meeting the diagnostic criteria for PTSD.

Indeed, the present finding is consistent with other studies demonstrating that induced anxiety enhances spatial WM (Duncko et al. 2007; Yuen et al. 2009; Moriya and Sugiura 2012). The finding that spatial WM is improved by threat is intuitively appealing from an evolutionary standpoint, as facilitation of visuospatial WM would allow accurate detection of stimuli during a dangerous situation and thus promote survival. This idea could help explain why anxiety improves performance at both encoding and retrieval: when reencountering a threatening environment it is of extra importance to remember the prior locations of potential threats. In line with this suggestion, prior research has demonstrated that threat increases aversive prediction error signal in the ventral striatum, indicating that anxiety may bias the predictive learning of threats to promote survival (Robinson et al. 2013a). In the context of anxiety disorders, however, it may be that these responses are exaggerated and/or perpetual, thus contributing to the maintenance of an anxious state (Liberzon and Abelson 2016); this proposal is in line with the finding that visual working memory capacity increases as trait anxiety increases (Moriya and Sugiura 2012). However, future research would benefit from further elucidating the differences between adaptive and maladaptive responding, as some research suggests anxiety disorder patients exhibit impairments in visuospatial short-term memory performance (Jelinek et al. 2006; O'Toole et al. 2015).

### Face recognition

In line with predictions, accuracy and confidence in the face recognition task were significantly greater when information was encoded under conditions of safety compared with threat. This indicates that anxiety impairs the encoding of faces, correspondingly reducing accuracy and confidence at recognition, and is in line with meta-analytic results from forensic studies that suggest induced anxiety at the time of encoding impairs person identification (Deffenbacher et al. 2004). This is likely due to anxiety during encoding degrading the quality of the memory representation; meaning information is not as accurately stored, therefore impairing retrieval and making it more effortful. Despite some previous studies demonstrating that face recognition is impaired by induced anxiety at retrieval (Attwood et al. 2015; Moon et al. 2016) our results do not substantiate this finding—when faces were encoded under conditions of safety, threat at retrieval did not impair performance. This is likely because the memory representation has already been successfully laid down during safe-encoding, and is therefore robust enough to withstand the impact of threat at retrieval.

The finding that anxiety impairs the encoding of faces has important implications. For example, in the context of eyewitness testimonies, impaired encoding and consequently identification of faces as a result of anxiety could have damaging consequences with regards to identifying criminals. Moreover, problems learning faces due to anxiety could help explain why individuals with anxiety disorders often have difficulties with social interactions, such as avoiding face-to-face exchanges. Notably, however, the current findings must be interpreted with caution as the face recognition task used a mixture of fearful, neutral, and positive faces. It may be that threat interacts with expression valence: during conditions of threat, negative faces may facilitate perception and encoding, and consequently retrieval. Future studies with higher power to explore valence effects, and a greater number of emotional face stimuli, should aim to investigate this.

### Verbal recognition

Inconsistent with predictions, there was no significant effect of anxiety, at encoding or retrieval, on accuracy or confidence in the verbal recognition task. Based on previous research demonstrating verbal WM performance is impaired by threat at low-levels of cognitive load, but unimpaired at high-levels (Vytal et al. 2013; Patel et al. 2016), it may be that the verbal recognition task was sufficiently demanding to protect against the impact of threat. However, it must be noted that most participants performed close to/at ceiling, suggesting the task was relatively easy. Indeed, it has been demonstrated that induced anxiety impairs WM performance only when the task is sufficiently demanding (Oei et al. 2006), this suggests that our verbal recognition task may not have been demanding enough to reach the threat-impairment threshold. Moreover, it is possible that threat had an effect on verbal recognition performance, but that this was masked due to the poor measurement properties of the task. This possibility is supported by the finding that participants were significantly faster when retrieving information under conditions of safety compared with under threat (see Supplemental Information).

### Associative memory

Again, contrary to hypotheses, results indicated that threat had no effect on associative memory, as Bayesian analysis enabled us to decisively accept the null hypothesis that threat had no effect at either encoding or retrieval. This suggests that episodic memories are not preferentially remembered when encoded under threat, and are preserved even when anxious at retrieval.

Notably however, this null-finding contradicts previous research that suggests induced anxiety, including threat of shock, impairs associative memory (Bisby and Burgess 2013; Guez et al. 2016). Differences in findings may partly be due to the current study's experimental setup. For instance, research has indicated that the impact of anxiety on explicit memory is dependent on the time interval between encoding and retrieval (Mitte 2008). This may help explain why our results were discrepant with previous findings (e.g., Bisby and Burgess 2013; Guez et al. 2016), as in the present study the time interval was around 5 min, whereas in Bisby and Burgess's (2013) study there was a delay of 24-h. It may be that the time interval between the encoding and retrieval stage of the associative memory task was not long enough to mimic true episodic memory. Additionally, in Bisby and Burgess's (2013) study, conditions of threat and safety were alternated on a trial-by-trial basis, in contrast to whole blocks of threat versus safe in the present study. This indicates that associative memory may be more likely to be impaired during shorter, more acute periods of threat compared with sustained periods.

### Summary of findings

The reasons behind this pattern of results are at present only speculative, however it has been suggested that worry (a cognitive dimension of anxiety) specifically interferes with phonological, rather than visuospatial, processes as they share neural resources (Vytal et al. 2012, 2013). Importantly however, worry is thought to be readily amenable to top-down control, suggesting that task-relevant goals can take precedence over worry (Ochsner and Gross 2005; Kalisch et al. 2006; Moran 2016). This may help explain why our results found no impact of threat on verbal short-term recognition and associative memory performance, both of which relied predominantly on phonological processing. Expressly, during the tasks that were primarily supported by verbal processing, off-task worry may have been suppressed, thus reducing task interference and leaving memory performance unimpaired.

In comparison, the finding that threat enhanced visuospatial working memory may confer an evolutionary advantage. Anxiety may increase perceptual sensitivity to help individuals better detect and remember the location of stimuli during threatening situations, helping to promote survival. This is in line with the idea that anxious arousal—a physiological response to threat (distinct from worry) that increases heart rate, blood pressure, etc.—primes processes that aid survival (e.g., Lang et al. 1998). In anxiety pathology, however, this may be an exaggerated and perpetual response, which contributes to the maintenance of an anxious state. Furthermore, impairment in the encoding of faces during threat may be because perceiving, recognizing, and encoding facial expressions involves a complex neural network (Palermo and Rhodes 2007) that may be easily disrupted by anxious arousal, which, in contrast to worry, is thought to specifically impact visual processing (Vytal et al. 2013). Moreover, while directing attention toward facial stimuli and registering whether expressions are threatening is important in the context of defensive readying, committing to memory the more fine-grained information needed to identify a face is unlikely to be a priority when encountering threat.

**Figure 5.**
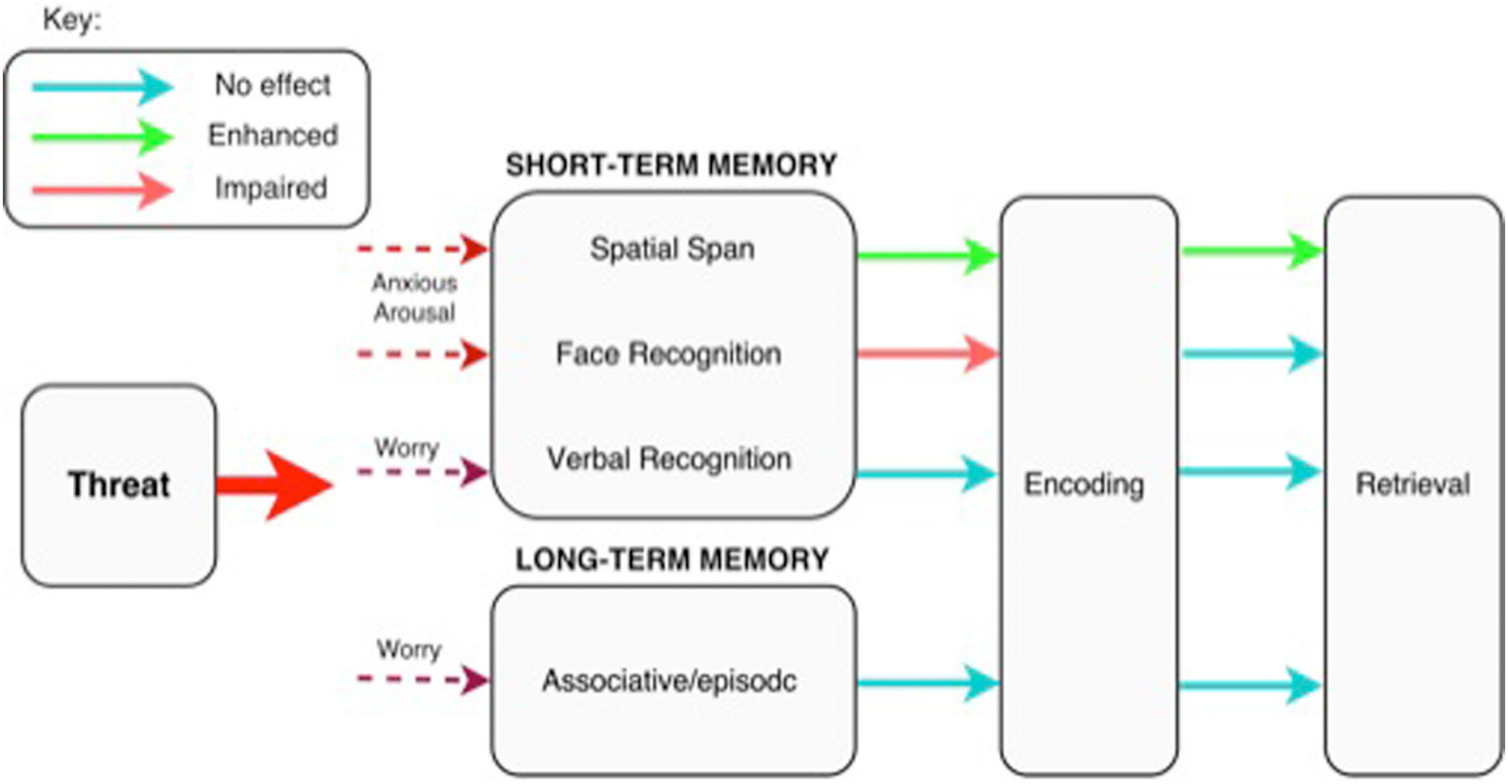
Model of overall findings. Box diagram representing the impact of threat (see “Key”) on memory performance at encoding and retrieval. For example, threat at encoding, but not retrieval, impaired short-term facial recognition performance. The dashed lines represent the hypothesized dissociable impact of threat-induced anxious arousal and worry on different memory processes.

The proposal that distinct facets of anxiety—namely “cognitive worry” versus “anxious physiological arousal”—differentially impact memory processes is consistent with the emerging “two systems” view of anxiety. This framework suggests that anxiety-related behavioral responses and their associated physiological changes are underpinned by neural circuits that are separable to those sub-serving the conscious emotional expression of anxiety (LeDoux and Pine 2016). Taken together, our findings indicate that threat has a dissociable impact on memory modality (verbal/phonological versus visual/spatial), stage (encoding versus retrieval), and time course (short- versus long-term) (Fig. 5).

### Strengths and limitations

A major strength of the current study is its novel use of threat of shock to dissociate the impact of anxiety on the encoding and retrieval stages of a variety of memory processes. An additional methodological advantage is the within-subjects design, which increased statistical efficiency, and allowed participants to serve as their own controls. Moreover, the use of healthy individuals bypassed some of the challenges associated with anxiety patient studies (e.g., comorbidities, time of disorder onset). However, it is important to acknowledge limitations beyond those already discussed. First, as the tasks differed on several dimensions, it is difficult to clearly interpret observed effects. Therefore, in order to more formally test the proposition that visuospatial versus verbal memory processes are differentially impacted by anxiety, future research would benefit from a study paradigm that uses similar/identical memory tasks (for both long- and short-term) but with either visual or verbal stimuli. Additionally, the tasks can be criticized for lacking ecological validity, as for the most part they are unlike tasks that would be encountered in real-life scenarios. For instance, the associative memory task, although better than pairwise associative memory tasks, failed to assess the spatiotemporal subcomponent (i.e., “when”) of episodic memory (Pause et al. 2013; Zlomuzica et al. 2014). To increase ecological validity, findings could be replicated using, for example, virtual–reality paradigms with real-life objects and scenarios. Lastly, while threat of shock is a robust and reliable way of inducing anxiety, it must be noted that it may take more extreme levels of threat (e.g., combat/warzone situations) to see an impact on associative and verbal recognition memory, and that the current findings may not generalize to other anxiety manipulations. In particular, as these manipulations will differ in terms of cortisol response times, the extent to which the anxiety induction carries over to the experimental task, and physiological versus cognitive dimensions of anxiety (e.g., CO_2_ inhalation versus evaluation stress).

### Conclusions

The present results demonstrate a clear effect of threat of shock on facial recognition and visuospatial working memory performance: with threat impairing the encoding of faces, and enhancing the encoding and subsequent retrieval of spatial locations. In contrast, threat left verbal short-term recognition and associative memory performance ostensibly intact. While a conclusive model of anxiety's relationship to memory is premature without further research, this pattern of findings indicates that threat has a domain-specific rather than domain-general impact on short- and long-term memory. Our findings suggest that memory processes that rely predominantly on verbal processing are less susceptible to the impact of threat of shock-induced anxiety, compared with visual/spatial memory processes. Overall, this pattern might represent a compromise, whereby threat-relevant (e.g., spatial) learning is enhanced, but at a cost to other (e.g., face recognition) learning processes. Nevertheless, our explanations as to why some domains are impaired, some augmented and some unperturbed are at present speculative and warrant further research.

This sheds light on nonpathological responses to threat in healthy individuals, and may further provide an insight into how memory is disrupted in anxiety disorders, which can help tailor treatment. Future research would benefit from expanding these findings into anxiety disorder patients to delineate the differences between adaptive and maladaptive responding, and so enhance understanding of the relationship between anxiety and memory.

## Materials and Methods

### Participants and screening

Participants were recruited from the Institute of Cognitive Neuroscience Subject Database. As preregistered, a sample size of 78 participants was required to achieve 80% power (at α = 0.05, for effect size *d* = 0.65: effect size based on similar experimental design (Balderston et al. 2017a), and was estimated using a power calculation conducted in G*Power 3.7.9, powering the analysis to detect a within-between interaction in a mixed measures design. A total sample size of 86 participants (50 female, 36 male; age range 18–49, *M* = 24.7, SD = 6.36) completed the facial and verbal recognition tasks, and the spatial working memory task, and 78 of these participants (48 female, 30 male; age range 18–49, *M* = 24.6, SD = 6.45) completed the associative memory task. Participants provided written informed consent, and received up to £15 as remuneration. The UCL Research Ethics Committee approved the study (Project ID number: 1764/001).

Prior to completing the study participants’ eligibility was assessed via a telephone-based screening interview. Exclusion criteria included; significant current/past medical or psychiatric illness; significant physical abnormality (e.g., history of cardiac or respiratory problems, including asthma); history of bipolar disorder or schizophrenia in a first-degree relative; history of alcoholism or drug dependence; recent use of illicit drugs; impaired or uncorrected vision or hearing; and pregnancy.

### Design

For three out of four tasks threat of shock during encoding and retrieval was manipulated within-subjects. Participants completed three different memory tasks (verbal recognition, face recognition, spatial span—see “Memory tasks”) in which information was encoded during safe versus threat, and subsequently retrieved during safe versus threat. This gave rise to a 2(encoding: safe versus threat) × 2(retrieval: safe versus threat) design with four groups: threat/threat, threat/safe, safe/threat, safe/safe.

The fourth task (associative memory) had a mixed factorial design to reduce task length: threat of shock during memory encoding was manipulated within-subjects (with participants encoding information during safe versus threat), and threat of shock during memory retrieval was manipulated between-subjects (information retrieved during safe or threat conditions).

### Materials

#### Anxiety manipulation

Threat of shock was administered according to a standardized procedure (e.g., Robinson et al. 2013c) using a digitimer DS5. The shocks were administered to the participant's wrist of their nondominant hand. During the encoding and retrieval phases of each task participants were told they were either “safe from shock” (safe condition) or “at risk of shock” (threat condition). During the threat condition shocks were delivered at a pseudorandom time point, no more than four times during each task. All shocks were given during the inter-trial intervals; this helped ensure that the shocks themselves did not affect performance.

Before the main experiment began, in order to minimize the risk associated with electric shocks, there was a shock “work-up” procedure. The shock work-up procedure started by administering shocks at a very low level, and shock intensity was successively increased to a level that was appropriate for each participant. To determine what level was appropriate, participants rated how uncomfortable they found each shock on a scale of 1 (not at all) to 5 (very); once a rating of at least 4 out of 5 had been reached shock intensity was not increased any higher. Once an appropriate level had been determined, the participants completed the four tasks (see below) under conditions of alternating “threat” or “safe.” As a manipulation check participants reported retrospective ratings of anxiety (from 1 “not at all anxious,” to 9 “extremely anxious”) for the threat and safe conditions. Due to the tasks’ setup, rating was done either at the end of each experimental block, or at the end of the task (see “Memory tasks” for details).

#### Memory tasks

The four memory tasks were run on the computer and completed under both threat and safe conditions. Tasks were programmed in MATLAB R2015b, and run using the Psychophysics Toolbox extension (Version 3; Brainard 1997; Pelli 1997; Kleiner et al. 2007) and the Cogent Toolbox (Cogent, http://www.vislab.ucl.ac.uk/Cogent/). Task order was counterbalanced across participants to help control for any effect of shock desensitization over time. For each task, separate stimuli were used for each of the four conditions (safe/safe, safe/threat, threat/safe, threat/threat) so responses were not influenced by habituation to stimuli. Additionally, for all tasks, the order in which the conditions were presented was counterbalanced across participants. During each of the tasks, when participants were at threat of shock an onscreen warning was given (“Warning you are now at risk of shock!”) and the screen displayed a red border for the duration of the threat period. When participants were safe from shock the onscreen message notified them (“You are now safe from shock”), and the screen displayed a blue border for the duration of the safe period. The four tasks are outlined below.

For the first three tasks outlined below, there are four blocks (one per condition) where participants encode and immediately retrieve the information under conditions of either threat or safety. For the fourth task outlined below (associative memory task), all information was encoded (under one block of threat, one of safe) and subsequently retrieved following a 5 min time interval (under threat or safe conditions). For task schematics see Supplemental Information.

##### Spatial span task

(To assess visuospatial working memory) In each trial, participants saw nine gray boxes on the screen, which changed color (color changed every block) in a variable sequence. To begin, two gray boxes changed color, and to end nine changed color: giving a total of nine trials per block. Participants were asked to remember the order in which the boxes changed color. During the retrieval phase (which immediately followed encoding) they were presented with the same nine boxes with numbers in, and had to indicate the correct sequence by pressing the corresponding numbers on the keyboard. The sequence in which the squares changed color varied each trial. The dependent variable was the total number of correct responses. There were a total of four blocks (one per condition), with nine trials per block. Before both the encoding and retrieval phases of each trial, an onscreen message notified participants if they were safe or at risk, and the screen either displayed a red or blue border. Participations rated their overall anxiety retrospectively (nine-point scale), for both the safe and threat conditions, at the end of the task. The total time to administer the task was ∼20 min.

##### Modified verbal recognition test

(To assess verbal short-term memory) Participants were shown a list of 10 neutral words, presented successively onscreen in a random order for 1300 msec each, and asked to try and remember the words that they saw. Participants were notified as to whether they were safe or at risk. Following the encoding phase, participants were presented with words that were either from the previously seen list or else unseen distractor words (18 words total). They indicated (with a “yes” versus “no” response; left and right computer arrow keys, respectively) whether they had seen the word previously or not. This retrieval phase was preceded by an onscreen message indicating whether participants were safe or at risk of shock. Participants rated their anxiety at the end of each encoding and retrieval phase (nine-point scale), as well as how confident they were in their responses from 1 (not at all confident) to 9 (extremely confident) at the end of each retrieval phase. The dependent variables were (per condition): average reaction time (seconds) (see Supplemental Information), total number of correct responses, and overall confidence in responses. There were four blocks in total (one per condition), and new word stimuli were used in each block. It took ∼10 min to administer the task.

##### Face recognition

Participants viewed 12 faces (6 male, 6 female; 6 black-ethnicity, 6 white-ethnicity; 4 fearful, 4 neutral, 4 happy), presented onscreen for 2500 msec each, and were asked to try and remember the faces they had seen. Prior to viewing these faces they were informed if they were safe or at risk of shock. Following the encoding stage, participants were shown another set of 24 faces (12 previously seen, 12 unseen: equal numbers of male/female/black/white/fearful/neutral/happy) and asked to indicate whether they had seen each face before or not (with a “yes” versus “no” response; left and right computer arrow keys, respectively). During this retrieval phase participants were told that they were either safe or at risk. The dependent variables were (per condition): average reaction time (seconds) (see Supplemental Information), total number of correct responses, and overall confidence in their answers. There were four blocks (one per condition) and new sets of 24 face stimuli were used per block. Faces were presented in a random order during the encoding and retrieval phases. Face stimuli were sourced from the Chicago Face Database (Ma et al. 2015), and displayed no unusual distinguishing features (e.g., beard, glasses). Participants rated their anxiety at the end of each encoding and retrieval phase, and the confidence in their answers at the end of each retrieval phase. The total time to administer the task was ∼15 min.

##### Associative memory task

(To assess associative memory; Horner and Burgess 2014) Participants learned events composed of three elements (locations, people, objects). Locations and objects were common places/items (e.g., kitchen, toothbrush), and the people were celebrities. For each event, participants were presented with three words, appearing onscreen simultaneously (for 6 sec), and were instructed to imagine the three elements interacting together as vividly as possible. A new event was presented each trial. During the retrieval phase, each trial consisted of a cue (e.g., a person), and participants were asked to select the associated element (e.g., the location) from five other elements of the same type taken from different events. All possible associations were tested (e.g., location–person), in both directions, resulting in six retrieval trials per event. The dependent variable was whether associations were correctly retrieved or not. The encoding stage contained a block of threat and a block of safe, counterbalanced across participants. Items were retrieved under either conditions of threat or safety, and this was counterbalanced across participants. As previously, during the encoding and testing phases, an onscreen message informed participants whether they were safe or at risk, and the screen turned blue or red respectively. Participants gave retrospective anxiety ratings (9-point scale) at the end of the task. The total time to administer the task was ∼55 min.

### Procedure

Prior to the experimental session, participants were emailed details of the study. If participants were interested in taking part, their eligibility was assessed via a telephone-screening interview. This included obtaining demographic information, and a brief medical and psychiatric history. If participants were eligible (see “Participants and screening”), they were invited to an experimental session at the Institute of Cognitive Neuroscience, 17 Queen's Square, London, WC1N 3AZ.

To begin, the experimenter explained the nature of the experiment and participants were given the opportunity to ask questions. They then read the study information sheet, which explained the purpose of the experiment, the confidentiality and anonymity of results, and their right to withdraw. Participants gave written informed consent. Once consent had been obtained, the STAI and Raven's Matrices were administered (see Supplemental Information). Following this, the “shock work-up” procedure was conducted to determine an appropriate shock level. The memory tasks were then completed on the computer; task order was counterbalanced across participants. Each experimental session lasted ∼2 h.

### Analysis

For all analyses, standard frequentist and Bayesian repeated-measures ANOVA models were used (detailed below). Frequentist tests were conducted in SPSS (Version 22.0) and Bayesian analyses were run in JASP (Version 0.7.5.5), using the default prior (Rouder et al. 2012; Love et al. 2015). Frequentist tests produced *F*-statistics, *P*-values (α = 0.05) and effect sizes, and Bayesian ANOVAs generated Bayes Factors (BF_10_)^1^ for models of interest relative to a null model (main effect of subject). For Bayesian analyses, the model with the highest BF_10_ compared with the null was chosen as the “winning” model (BF_10_ <1 is evidence in favor of the null). The success of one model over another was calculated by dividing the larger BF_10_ by the smaller (>0 indicates the model is better than the comparison). To interpret comparisons, labels were assigned ranging from: anecdotal (1–3), to substantial (3–10), to strong (10–30) to decisive (>100) (Jeffreys 1998). Models containing interactions included the main effect of each component of the interaction. Inference was restricted to effects that were confirmed by both frequentist and Bayesian statistics.

Before analyses, the data was inspected for violations of the normality assumption. Violations, and the transforms applied, are described in the Results section. In short, data were either cubed or squared, depending upon whichever brought data closest to normality.

To confirm that threat of shock successfully induced anxiety, an average of participants’ retrospective anxiety ratings during safe and threat for each task was calculated. Subsequently, Wilcoxon signed-rank tests were run as in each case the data was non-normal, as well as Bayesian paired-samples *t*-tests.

For the facial recognition and verbal recognition tasks separate 2(encoding condition: threat versus safe) × 2(retrieval condition: threat versus safe) repeated-measures ANOVAs were constructed to examine the effect of threat on: proportion correct (0–1), reaction times (seconds), and confidence (1–9). The same model was constructed to assess the effect of threat on proportion correct (0–1) during the spatial span task. Proportion correct is the primary variable of interest reported in the current paper; analyses of confidence ratings are presented for completeness, however analyses of reaction times are provided in a Supplemental Information.

For the associative memory task a 2 × 3 × 2 mixed-measures ANOVA was run, with encoding condition (threat versus safe) and cue type (location versus person versus object) as within-subjects factors, and retrieval condition (threat versus safe) as the between-subjects factor.

All data and task scripts are available online (osf.io/zjpm2).

## Supplementary Material

Supplemental Material

## Footnotes

1 In some subsequent analyses, to make large BF_10_ values more interpretable, the natural log of the BF_10_ values is reported (logBF_10_).
