# Supplementary material for: The impact of threat of shock-induced anxiety on memory encoding and retrieval
Source: Learn Mem. 2017 Oct;24(10):532–42. doi: 10.1101/lm.045187.117 (PMC5602344; doi:10.1101/lm.045187.117)
Supplement: Supplemental Material [file supp_24_10_532__index.html]

Supplemental Material 

# The impact of threat of shock-induced anxiety on memory encoding and retrieval

## Supplemental Material

- SupplementalMaterial.docx
