# Supplementary material for: The impact of threat of shock-induced anxiety on memory encoding and retrieval
Source: Learn Mem. 2017 Oct;24(10):532–42. doi: 10.1101/lm.045187.117 (PMC5602344; doi:10.1101/lm.045187.117)
Supplement: Supplemental Material [file supp_24.10.532_SupplementalMaterial.docx]

**Supplement**

**Results**

**Verbal Recognition Task**

*Reaction Time*

Reaction time scores across all conditions were non-normal and so a logarithmic transformation was applied prior to analysis, to give a more normal distribution. No significant main effect of encoding condition was found, *F*(1,85)=0.33, *p*=.570, $\eta_{p}^{2}$=.004. There was a significant main effect of retrieval condition, *F*(1,85)=30.99, *p*<.001, $\eta_{p}^{2}$=.267, indicating that participants were significantly faster when retrieving information under conditions of safety (*M*=0.462, *SD*=0.220) compared to under threat (*M*=0.548, *SD*=0.255). There was no significant interaction between encoding and retrieval conditions, *F*(1,85)=0.42, *p*=.517, $\eta_{p}^{2}$=.005.

Bayesian analysis revealed the winning model to be one including the main effect of retrieval only (logBF_10_=14.15). This model was substantially (7.4 times) better than a model additionally including the main effect of encoding (logBF_10_=12.1), and strongly (38.2 times) better than the encoding × retrieval interaction model (logBF_10_=10.49).

**Face Recognition Task**

*Reaction Time*

Reaction time scores across all conditions were non-normal and so a logarithmic transformation was applied prior to analysis to give a more normal distribution. No significant main effect of encoding condition was found, *F*(1,85)=2.52, *p*=.116, $\eta_{p}^{2}$=.029. However, there was a significant main effect of retrieval condition, *F*(1,85)=11.45, *p*=.001, $\eta_{p}^{2}$=.119, indicating that participants were significantly faster when retrieving information under conditions of safety (*M*=0.541, *SD*=0.273) compared to under threat (*M*=0.588, *SD*=0.298). This effect was qualified by a significant interaction between encoding and retrieval conditions, *F*(1,85)=5.35, *p*=.023, $\eta_{p}^{2}$=.059.

Simple main effects analyses revealed that safety during encoding induced significantly faster reaction times (*M*=0.505, *SD*=0.255) compared to threat during encoding (*M*=0.577, *SD*=0.335), when information was retrieved under safe, *F*(1,85)=7.14, *p*=.009, $\eta_{p}^{2}$=.077. In contrast, there was no significant difference in reaction times between safety during encoding (*M*=0.609, *SD*=0.395) and threat during encoding (*M*=0.567, *SD*=0.244), when information was retrieved under threat, *F*(1,85)=0.26, *p*=.614, $\eta_{p}^{2}$=.003. Additionally, reaction times were significantly faster at safety during retrieval (*M*=0.505, *SD*=0.255) compared to threat during retrieval (*M*=0.609, *SD*=0.395) when information was encoded under safe, *F*(1,85)=22.18, *p*<.001, $\eta_{p}^{2}$=.207. However, there was no significant difference between reaction times at threat during retrieval (*M*=0.577, *SD*=0.335) compared to safety during retrieval (*M*=0.567, *SD*=0.244), when information was encoded under threat, *F*(1,85)=0.53, *p*=.469, $\eta_{p}^{2}$=.006. These differences can be seen in Figure 8.

Bayes factor analysis revealed the winning model to be one comprising only the main effect of retrieval (BF_10_=27.99), however this was only anecdotally (1.5 times) better than a model including the encoding x retrieval interaction (BF_10_=18.88).

**Associative Memory Task**

*Randomisation check*

As the retrieval condition was between-subjects, post-hoc analyses were run to ensure participants in the two retrieval conditions did not differ significantly from one another (refer to Supplemental_Methods.pdf for further details of the measures). For normally distributed variables independent-samples t-tests were conducted, with retrieval condition as the independent variable. The ‘age’ variable was unable to be normalised, therefore the non-parametric Mann-Whitney U test was conducted on the untransformed data. The total state anxiety scores were normalised by applying a natural logarithmic transformation to all values before analysis, and a ‘reflect and logarithmic’ transformation (Laerd Statistics, 2015) was used to normalise the total Raven’s Matrices Scores. Chi-squared tests were run on categorical variables: gender, English as first language (yes vs. no), and whether or not participants had done a threat of shock (ToS) study before (yes vs. no).

As seen in Table 1, there were no significant group differences in: age, total Raven’s Matrices scores, trait levels of anxiety, gender, English as a first language, and prior ToS experience, with the smallest p value being .070. State anxiety (measured using the STAI, which was administered before participants completed the memory tasks) did significantly differ across groups, *p*=.010, with participants in the safe retrieval condition exhibiting significantly greater state anxiety scores (*M*=35.88, *SD*=9.27) compared to those in the threat retrieval condition (*M*=30.41, *SD*=8.39).

Table 1. Randomisation Check Results.

*Means (and Standard Deviations) of key variables, and outputs for statistical tests comparing these across conditions*


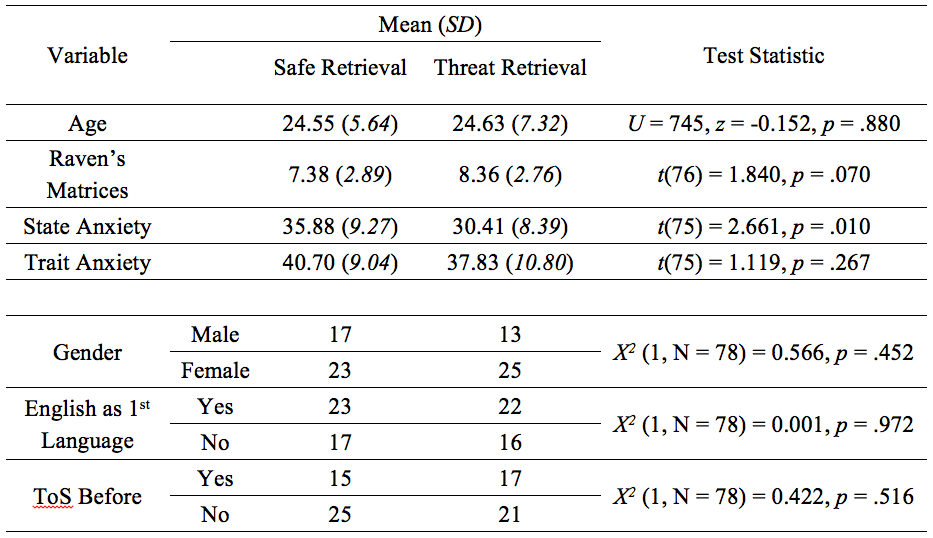


As there was a significant difference in state anxiety between the safe retrieval and threat retrieval groups (*p*=.010), all ANOVAs were repeated, entering state anxiety scores as a covariate. This exploratory analysis revealed a broadly similar pattern of results: frequentist statistics still found a significant three-way interaction between encoding condition, cue type, and retrieval condition, however the significant main effect of cue type no longer held (Huynh-Feldt correction applied), *F*(1.86, 137.90)=1.652, *p*=.197, $\eta_{p}^{2}$=.022., while Bayesian analysis still found the winning model to be the null.

**Safe Encoding**

**Temporal analysis**

Including the within-subject threat ratings in a 3 (time: 1,2,3) by 2 (condition: threat safe) ANOVA reiterated the main effect of threat (F(1,85)=248,p<0.001) but also showed a main effect of time (F(2,170)=9.2,p<0.001) driven by reduced ratings over time. However, this was seen equally across conditions (no threat by time interaction F(2,170)=1.3,p=0.28):


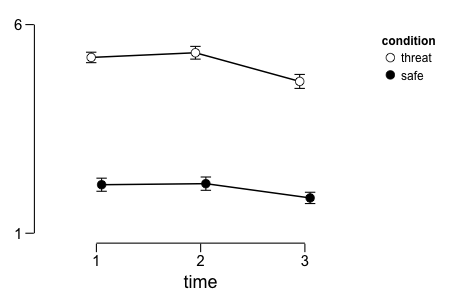


*Figure 4.* *Anxiety Ratings over time.* Anxiety rating declined over time, but equally for both conditions. Note that is includes the retrospective ratings following the fully within-subjects (facial, verbal and spatial) tasks only. Error bars represent standard error of the mean.

We counterbalanced task order to account for this possibility but can also re-run analyses adding task order as a covariate. Critically, the main effect of threat on self-report anxiety (F(1,158)=246,p<0.001), encoding during the facial task F(1,82)=13.2,p<0.001) and the interaction on the spatial task (F(1,80)=12.5,p<0.001) remained when counter-balanced task order was included as a factor in the analysis. Similarly, for both the verbal and associative memory tasks, the main effects and interactions of interest remained BF10<1, thereby continuing to favour the null (and rejecting the influence of threat on these tasks). In sum, this suggests that our inference is not influenced by a decline in the efficacy of our manipulation over time.

**Methods**

**Materials**

The State Trait Anxiety Inventory (STAI; e.g. Spielberger, Gorsuch, Lushene, Vagg, & Jacobs, 1983) was used to measure participants self-reported state and trait anxiety, to ensure levels did not confound results. Participants rated 20 items for trait anxiety (e.g. “I worry too much over something that really doesn’t matter”), and 20 items for state anxiety (e.g. “I am tense; I am worried”) on a 4-point scale from 1 (“Almost Never”) to 4 (“Almost Always”). Higher scores index greater anxiety. The STAI has good construct validity, test-retest reliability, and strong internal consistency: with *α* ranging from 0.86 to 0.95 (Spielberger et al., 1983).

Raven’s Progressive Matrices (e.g. Raven, 2000) were used as an index of intelligence, to ensure that levels did not confound results. Participants were given a shortened version of the test made up of 12 patterned diagrams, each with a part missing. Participants had to choose the correct part to complete the diagram from a set of eight options presented underneath. Answers were marked as either correct (1) or incorrect (0), and raw total scores (out of a maximum of 12) were used in subsequent analysis. Raven’s Progressive Matrices have been shown to be a reliable measure of intelligence: α ranging from 0.67 to 0.89 (de Abreu, Conway, & Gathercole, 2010; Rushton & Skuy, 2001).

**Task Schematics**


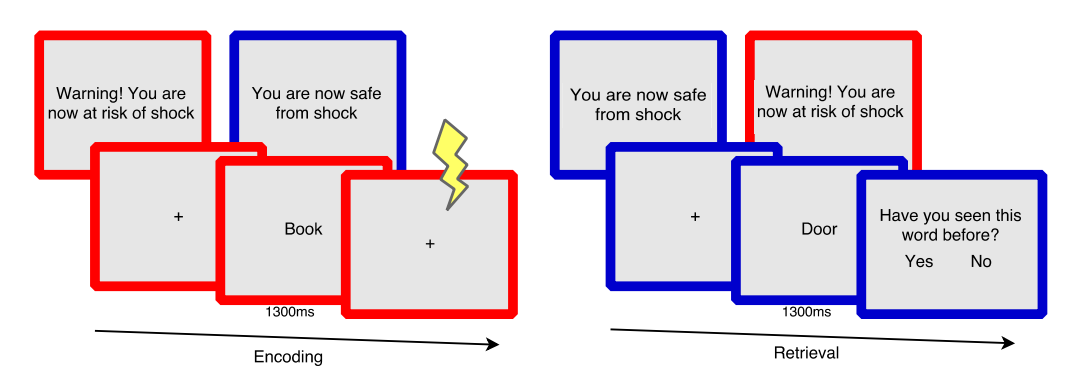


*Figure 2.* *Verbal Recognition Task Sequence.* Encoding and retrieval stages of the task were completed under both threat (red border; pseudorandom shocks administered) and safe (blue border)


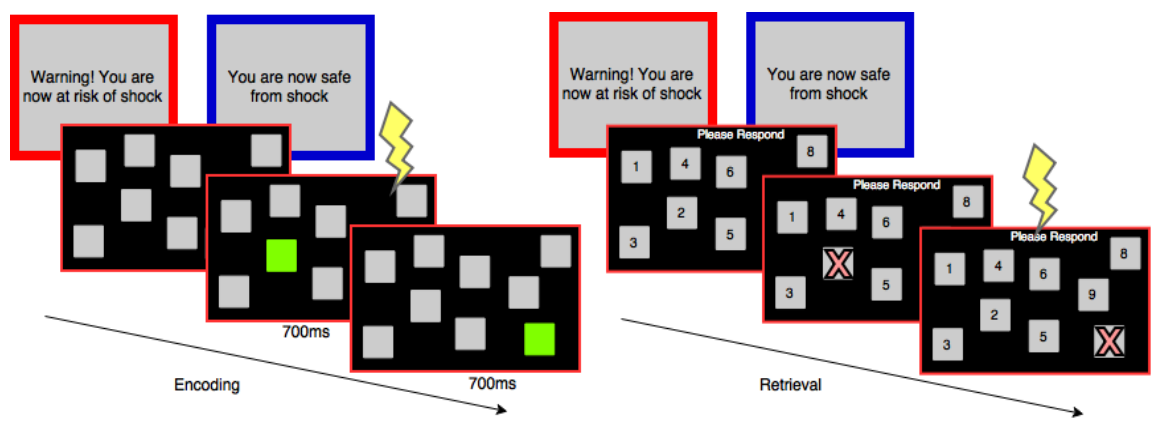


*Figure 3. Spatial Span Task Sequence.* Encoding and retrieval stages of the task were completed under both threat (red border; pseudorandom shocks administered) and safe (blue border).


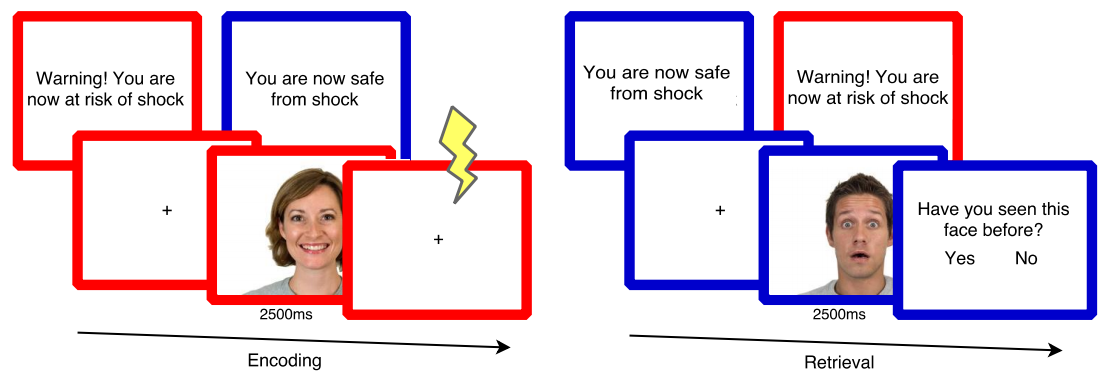


*Figure 4.* *Facial Recognition Task Sequence.* Encoding and retrieval stages of the task were completed under both threat (red border; pseudorandom shocks administered) and safe (blue border).


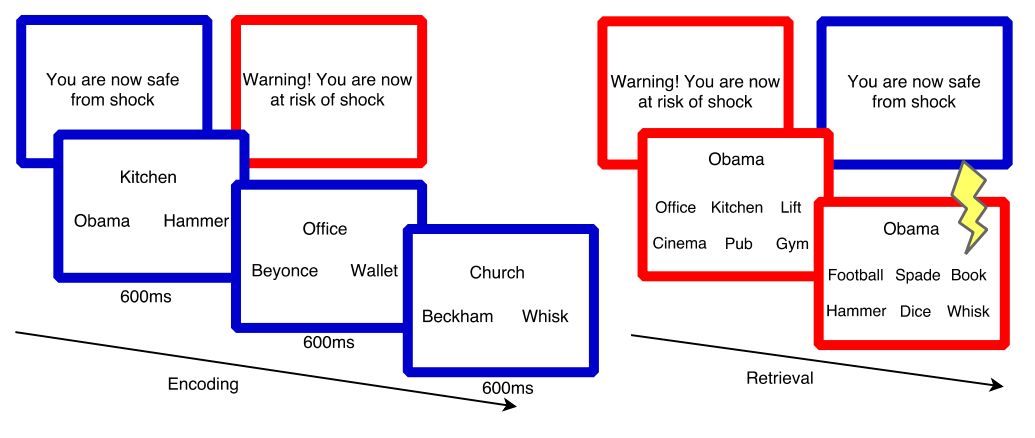


*Figure 4.* *Associative Memory Task Sequence.* The encoding stage of the task was completed under both threat (red border; pseudorandom shocks administered) and safe (blue border), and the retrieval stage under either threat or safe conditions.

**Additional plots**


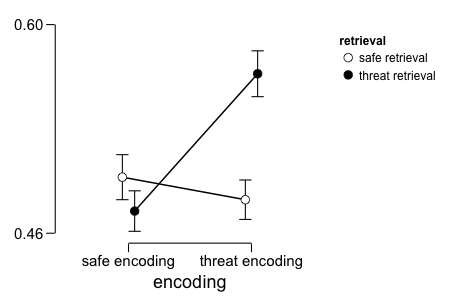


*Figure 5.* *Spatial Span task.* JASP plot of the encoding by retrieval interaction. Error bars represent standard error of the mean


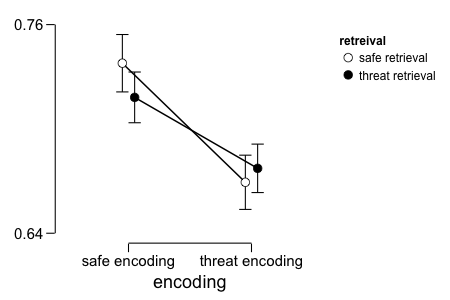


*Figure 6.* *Facial Recognition Task.* JASP plot of the encoding by retrieval interaction. Error bars represent standard error of the mean

.

**References**

de Abreu, P. M. J. E., Conway, A. R. A., & Gathercole, S. E. (2010). Working memory and fluid intelligence in young children. *Intelligence*, *38*(6), 552–561. doi:10.1016/j.intell.2010.07.003

Raven, J. (2000). The Raven’s progressive matrices: change and stability over culture and time. *Cognitive Psychology*, *41*(1), 1–48.

Rushton, J. P., & Skuy, M. (2001). Performance on Raven’s Matrices by African and White university students in South Africa. *Intelligence*, *28*(4), 251–265.

Spielberger, C. D., Gorsuch, R. L., Lushene, R., Vagg, P. R., & Jacobs, G. A. (1983). *Manual for the State-Trait Anxiety Inventory.* Palo Alto, CA: Consulting Psychologists Press.
